# Arterial hyperoxia and mortality in critically ill patients: a systematic review and meta-analysis

**DOI:** 10.1186/s13054-014-0711-x

**Published:** 2014-12-23

**Authors:** Elisa Damiani, Erica Adrario, Massimo Girardis, Rocco Romano, Paolo Pelaia, Mervyn Singer, Abele Donati

**Affiliations:** Anaesthesia and Intensive Care Unit, Department of Biomedical Sciences and Public Health, Università Politecnica delle Marche, via Tronto 10, 60126 Torrette di Ancona, Italy; Department of Anaesthesia and Intensive Care, University Hospital of Modena, Via del Pozzo 71, 41124 Modena, Italy; Bloomsbury Institute of Intensive Care Medicine, University College London, Gower Street, London, WC1E 6BT UK

## Abstract

**Introduction:**

The safety of arterial hyperoxia is under increasing scrutiny. We performed a systematic review of the literature to determine whether any association exists between arterial hyperoxia and mortality in critically ill patient subsets.

**Methods:**

Medline, Thomson Reuters Web of Science and Scopus databases were searched from inception to June 2014. Observational or interventional studies evaluating the relationship between hyperoxia (defined as a supranormal arterial O_2_ tension) and mortality in adult intensive care unit (ICU) patients were included. Studies primarily involving patients with exacerbations of chronic pulmonary disease, acute lung injury and perioperative administration were excluded. Adjusted odds ratio (OR) of patients exposed versus those not exposed to hyperoxia were extracted, if available. Alternatively, unadjusted outcome data were recorded. Data on patients, study characteristics and the criteria used for defining hyperoxia exposure were also extracted. Random-effects models were used for quantitative synthesis of the data, with a primary outcome of hospital mortality.

**Results:**

In total 17 studies (16 observational, 1 prospective before-after) were identified in different patient categories: mechanically ventilated ICU (number of studies (k) = 4, number of participants (n) = 189,143), post-cardiac arrest (k = 6, n = 19,144), stroke (k = 2, n = 5,537), and traumatic brain injury (k = 5, n = 7,488). Different criteria were used to define hyperoxia in terms of PaO_2_ value (first, highest, worst, mean), time of assessment and predetermined cutoffs. Data from studies on ICU patients were not pooled because of extreme heterogeneity (inconsistency (I^2^) 96.73%). Hyperoxia was associated with increased mortality in post-cardiac arrest patients (OR = 1.42 (1.04 to 1.92) I^2^ 67.73%) stroke (OR = 1.23 (1.06 to 1.43) I^2^ 0%) and traumatic brain injury (OR = 1.41 (1.03 to 1.94) I^2^ 64.54%). However, these results are limited by significant heterogeneity between studies.

**Conclusions:**

Hyperoxia may be associated with increased mortality in patients with stroke, traumatic brain injury and those resuscitated from cardiac arrest. However, these results are limited by the high heterogeneity of the included studies.

**Electronic supplementary material:**

The online version of this article (doi:10.1186/s13054-014-0711-x) contains supplementary material, which is available to authorized users.

## Introduction

Oxygen (O_2_) administration is the most widely prescribed therapy in critically ill patients and frequently represents a life-saving intervention. Since hypoxemia is generally viewed as deleterious and moderate levels of arterial hyperoxia as benign, health care practitioners are more likely to accept supranormal arterial O_2_ levels as this provides a wider safety buffer [1,2].

The use of supplemental O_2_ in various medical emergencies is supported by many guidelines [3-5]. One hundred percent O_2_ is commonly administered during cardiopulmonary resuscitation from cardiac arrest [6]. Normobaric hyperoxia is touted as a potential therapeutic strategy for patients with traumatic brain injury or stroke [7], with an underlying rationale of increased brain O_2_ delivery [8] and protection of the ischemic penumbra through inducing redistribution of blood from normal to ischemic areas [9]. However, these potential benefits must be weighed against the injurious effects of high-dose supplemental O_2_. In both animal and human studies there are reports of pulmonary toxicity [10-12], increased vasoconstriction with a fall in cardiac output [13], free radical-mediated damage to various organs [14], and a marked reduction in coronary blood flow and myocardial O_2_ consumption [15].

Clinical data regarding the relationship between arterial hyperoxia and outcome are contradictory. An association between arterial hyperoxia and mortality has been reported in disparate patient populations (mechanically ventilated [16], post-cardiac arrest [17], traumatic brain injury [18], stroke [19]) but not confirmed by other studies [20-23]. Therefore, the question whether exposure to supranormal arterial O_2_ tensions (PaO_2_) is safe in critically ill patients remains unanswered. We thus performed a systematic review and meta-analysis of studies describing the relationship between arterial hyperoxia and mortality in critically ill patients.

## Materials and methods

This report adheres to the Preferred Reporting Items for Systematic reviews and Meta-Analysis (PRISMA) standards for reporting systematic review and meta-analysis studies [24].

### Eligibility criteria

Observational (prospective or retrospective cohort or case-control studies) or randomized controlled trials (RCTs) investigating the relationship between arterial hyperoxia and mortality in critically ill patients were eligible for inclusion. Participants were required to be adult patients admitted to a critical care unit for any reason. We excluded studies involving patients with an acute exacerbation of chronic obstructive pulmonary disease (COPD) or acute lung injury (ALI). As hypoxemia is the main problem in these patients, studies on the impact of hyperoxemia were likely to be uncommon. We expected that studies on patients with COPD/ALI may have explored the effects of excessive O_2_ flow rather than those of arterial hyperoxemia. In addition, in these patients the PaO_2_ range defined as normal/acceptable could have been lower than that applied in patients with preserved respiratory function. Studies on surgical patients were excluded unless they were exposed to hyperoxia during a post-operative admission to a critical care unit. Hyperoxia was defined by the measurement of supranormal values of arterial partial O_2_ pressure (PaO_2_). For defining a condition of exposure to hyperoxia, any cutoff value of PaO_2_ and time of assessment were deemed acceptable. Studies where patients were defined as ‘hyperoxic’ solely on the basis of exposure to a predetermined increase in inspired O_2_ fraction (FiO_2_) were excluded if not guided by any assessment of PaO_2_. Patients not exposed to hyperoxia constituted the comparator group. The primary outcome of interest was hospital mortality from any cause.

### Search strategy

Studies were identified by searching Medline (PubMed), Scopus and Thomson Reuters Web of Science databases from their inception. The main search was run on 28 March 2014 and updated weekly until June 2014. The keywords ‘hyperoxia’, ‘hyperoxemia’, ‘arterial oxygen’, ‘oxygen saturation’, ‘critically ill’, ‘acutely ill’, ‘intensive care’, ‘critical care’, ‘mechanically ventilated’, ‘cardiac arrest’, ‘cardiopulmonary resuscitation’, ‘traumatic brain injury’, ‘head trauma’, ‘stroke’, ‘sepsis’, ‘septic shock’, ‘trauma’, ‘post-operative’, ‘post-surgery’, ‘cardiac failure’, ‘heart failure’, ‘myocardial infarction’, ‘shock’, ‘mortality’, ‘survival’, ‘death’, ‘outcome’ were typed in various combinations using Boolean operators. Queries were limited to those involving human subjects. The detailed search strategy applied to Medline (PubMed), and adapted for the other databases, is described in Additional file 1. Hand searches of reference lists of articles and relevant literature reviews were used to complement the computer search. Content pages of the main critical care medicine journals were hand-searched to find any relevant in-press articles. The search was not limited by language, but focused solely on articles published in peer-reviewed journals to enhance the methodological rigor of the studies examined and the conclusions drawn.

### Study selection

Two independent reviewers (ED and EA) screened all identified records (title and abstract) and performed the eligibility assessment of the selected full-text articles in an unblinded standardized manner. Disagreements were resolved through discussion or arbitration by a third reviewer (AD). Interobserver agreement was assessed by kappa statistics.

### Data extraction

Two independent investigators (ED and EA) extracted descriptive, methodological and outcome data from all eligible studies using a predefined data extraction form. Disagreements were resolved through consensus. The datasheet included study design (RCT, retrospective or prospective observational study, multicenter or single-center), country, years of enrollment, publication year, primary endpoint, sample size, mean age (as a continuous variable), gender distribution (as a percentage of males), category of critical illness (mechanically ventilated, post-cardiac arrest, traumatic brain injury, stroke, other), criteria for the definition of hyperoxia exposure (time of assessment, selection of the first/highest/worst/mean PaO_2_, cutoff value to define hyperoxia), prevalence of hyperoxia, overall in-hospital mortality, and prevalence of chronic cardiovascular and/or respiratory disease. Additional data were extracted for studies on post-cardiac arrest patients: average delay to return of spontaneous circulation (minutes); prevalence of initial shockable rhythm (ventricular tachycardia/fibrillation); prevalence of out-of-hospital cardiac arrest; prevalence of therapeutic hypothermia. Unadjusted outcome data (number of survivors and nonsurvivors to hospital discharge in hyperoxic and nonhyperoxic patients) and adjusted odds ratio (OR) (95% confidence interval) describing the association between hyperoxia exposure and mortality were extracted for calculation of effect size (ES). In-hospital mortality was chosen as the primary endpoint of our analysis since it was the outcome measure most frequently reported. When in-hospital mortality was not stated, we extracted data reporting the longest-term mortality available. The study authors were contacted to request additional information whenever a study did not report data necessary for calculation of the ES.

### Study quality assessment

The Newcastle-Ottawa Scale (NOS) for cohort studies was used to assess the quality of the included studies [25]. The item ‘representativeness of the exposed cohort’ was fulfilled if ≤10% of patients had been excluded because of missing data. The item ‘completeness of follow-up’ was fulfilled if ≤10% of patients had been excluded because of missing mortality data. For assessment of comparability of cohorts, two confounding factors were defined *a priori*: illness severity (as defined by any of the following severity scores: Acute Physiology and Chronic Health Evaluation (APACHE), Simplified Acute Physiology Score (SAPS), Sequential Organ Failure Assessment (SOFA), Injury Severity Score (ISS)) and FiO_2_. A study was considered to adequately control for each of these factors if it either demonstrated balance between groups for the confounder, or adjusted for it in the statistical analysis.

### Statistical analysis

Data were synthesized using meta-analytic methods [26,27], and statistically pooled by the standard meta-analysis approach, that is studies were weighted by the inverse of the sampling variance. Whenever possible, calculation of the ES of individual studies was based on the adjusted OR of the association between hyperoxia exposure and mortality as compared to nonexposure. When the authors reported results from more than one multivariate model, we extracted data deriving from either the model that included the maximum number of covariates, or the model that included severity of illness and FiO_2_. If the authors analyzed the unadjusted or adjusted association between mortality and increasing quartiles/quintiles/deciles of PaO_2_, we considered patients as ‘hyperoxic’ if they fell in the upper stratum. When adjusted data were not reported or PaO_2_ was analyzed as a continuous variable, unadjusted ORs were reconstructed from binary raw data (number of survivors/nonsurvivors in hyperoxia exposed/not exposed). When normoxia and hypoxemia were considered as two separate categories, only normoxic patients were included in the comparator group. Overall ES was expressed as OR and its corresponding 95% confidence interval (CI). The DerSimonian and Laird random effects model was used as a conservative approach to account for different sources of variation among studies. Forest plots were constructed to graphically represent the results. Q statistics were used to assess heterogeneity among studies. A significant Q value indicates a lack of homogeneity of findings among studies [26,27]. Inconsistency analysis *(I*^*2*^*)* statistics were then used to quantify the proportion of observed inconsistency across study results not explained by chance [28]. I^2^ values of <25%, 50% and >75% represent low, moderate and high inconsistency, respectively [28]. Sensitivity analyses were planned *a priori* to assess the impact of potential outliers (based on statistical significance of the standardized residuals) and sources of heterogeneity. Several variables were identified and their effects on outcome examined. Categorical variables were treated as moderators and the strength of the association between hyperoxia and mortality assessed and compared across subgroups formed by these moderators. Continuous variables were examined as covariates using random effects meta-regression. Meta-regression was performed to assess the effect of study quality (NOS score) on the calculated estimate. The presence of publication bias was investigated through funnel plots both visually and formally by trim and fill analysis and Eggers’s linear regression method [29]. A *P* value less than 0.05 was used to indicate statistical significance. All analyses were conducted using a computer software package (ProMeta Version 2, Internovi, Cesena FC, Italy).

## Results

**Figure 1:**
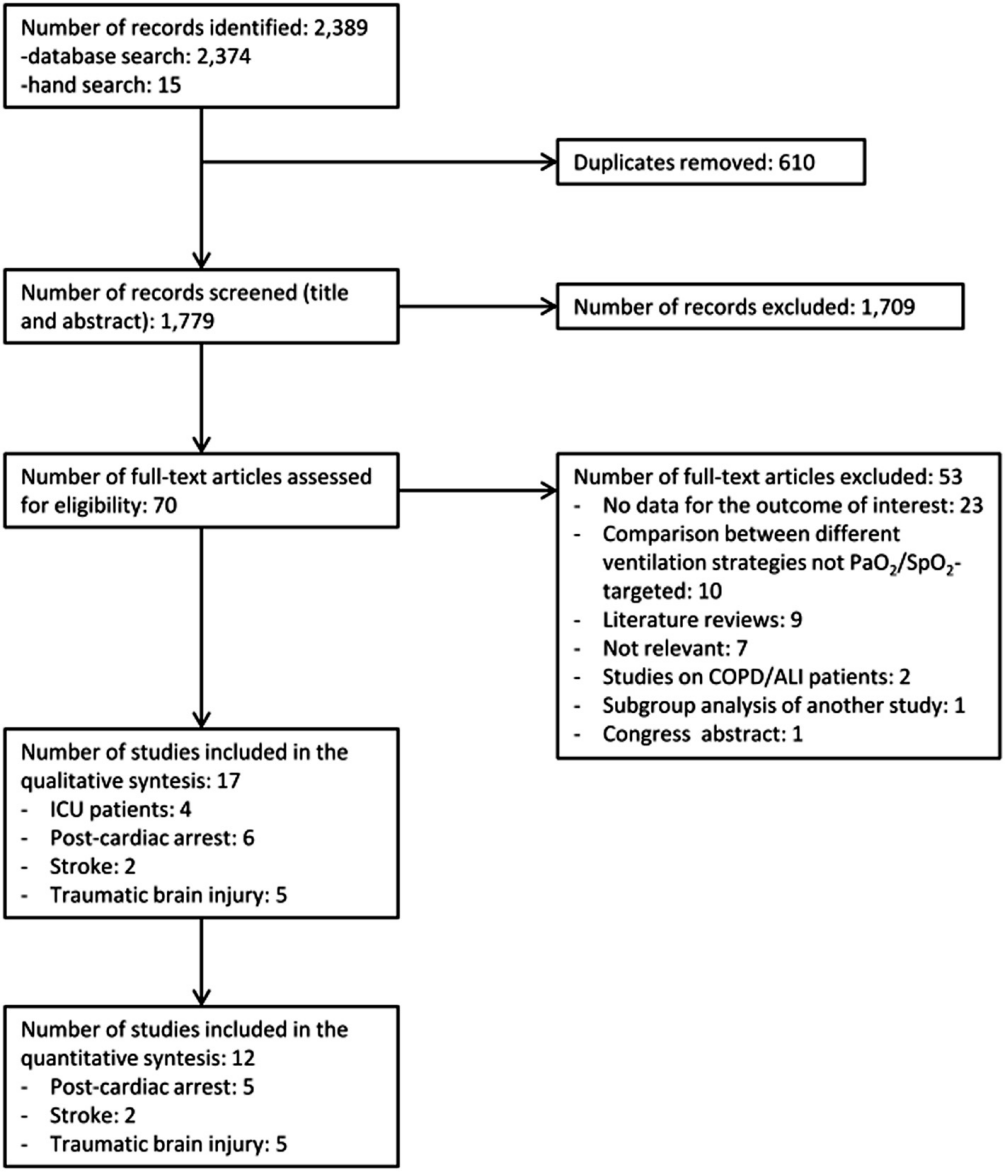
**Flow chart representing the selection process of the studies included in the qualitative and quantitative syntheses.**

From the 2,389 articles that were initially identified (Figure 1), 70 potentially relevant original articles were examined in full text (κ = 0.87 (95% CI, 0.85 to 0.90)). Seventeen studies eventually met our inclusion criteria (κ = 0.91 (0.88 to 0.94)). The study by Kilgannon *et al.* [30] was excluded as it was a subgroup analysis of the same patient population previously described by the same group [17]. For the study by Ihle *et al.* [31] we extrapolated outcome data related to the years 2010 to 2011, as the authors used the same database as Bellomo *et al.* [21] with study populations overlapping for the years 2007 to 2009. Two studies were identified in which hyperoxia exposure was defined on the basis of a peripheral O_2_ saturation (SpO_2_) >98% [2,32]; although it is questionable whether these patients were really hyperoxic to a significant degree, we decided to include these studies in the analysis as the reported time-weighted PaO_2_ values were above the upper normal limit of 100 mmHg in both cases [2,32].

The 17 studies identified were all published in English between 2008 and 2014 and involved different categories of critically ill patients [2,16-23,31-38]. Only four studies (24%) involved general populations of mechanically ventilated intensive care unit (ICU) patients [2,16,20,32]. Six studies focused upon patients resuscitated from cardiac arrest [17,21,31,35-37], five studies evaluated patients with traumatic brain injury [18,22,33,34,38], while two studies involved patients with stroke [19,23]. The main characteristics of the studies are reported in Table 1. Individual unadjusted/adjusted outcome data and variables included in the multivariable models are reported in Table 2. Study quality assessment is reported in Additional file 2.

**Table 1:** **Characteristics of the included studies**

**Study**	**Design**	**Country**	**Hyperoxia, definition**			**Comparator group**	**Outcome measure reported**
			**PaO**_**2**_ **/ABG**	**Time of assessment**	**Cutoff value**		
***Mechanically ventilated ICU patients***							
de Jonge *et al*. 2008 [16]	Retrospective cohort, multicenter	Netherlands	Worst PaO_2_	First 24 hours	≥120 mmHg (upper quintile)	PaO_2_ between 66 and 80 mmHg	In-hospital mortality
Eastwood *et al*. 2012 [20]	Retrospective cohort, multicenter	Australia, New Zealand	Worst PaO_2_	First 24 hours	>120 mmHg for unadjusted analysis; ≥305 mmHg (upper decile) for adjusted analysis	PaO_2_ <120 mmHg for unadjusted analysis; PaO_2_ between 75 and 85 mmHg for adjusted analysis	In-hospital mortality
Suzuki *et al.* 2013 [2]	Prospective observational cohort, single-center	Australia	Time-weighted SpO_2_	Whole period of mechanical ventilation	Time-weighted SpO_2_ >98%	Not exposed to hyperoxia	In-hospital mortality
Suzuki *et al*. 2014 [32]	Prospective before-after, single-center	Australia	*Conventional period:* oxygenation goals at the discretion of the attending physicians			*Conservative period:* SpO_2_ between 90 and 92%	28-day mortality
***Post-cardiac arrest patients***							
Bellomo *et al.* 2011 [21]	Retrospective cohort, multicenter	Australia, New Zealand	Worst PaO_2_	First 24 hours	≥300 mmHg	Normoxia	In-hospital mortality
Ihle *et al*. 2013 [31]	Retrospective cohort, multicenter	Australia	Worst PaO_2_	First 24 hours	≥300 mmHg	Normoxia	In-hospital mortality
Janz *et al.* 2012 [35]	Retrospective analysis of a prospective cohort study, single-center	USA	Highest PaO_2_	First 24 hours	≥300 mmHg^a^	Not exposed to hyperoxia	In-hospital mortality
Kilgannon *et al*. 2010 [17]	Retrospective cohort, multicenter	USA	First PaO_2_	First 24 hours	≥300 mmHg	Not exposed to hyperoxia	In-hospital mortality
Lee *et al.* 2014 [36]	Retrospective cohort, single-center	Korea	Mean PaO_2_	From return of spontaneous circulation to the end of therapeutic hypothermia	≥156.7 mmHg (upper quartile)	PaO_2_ between 116 and 134.9 mmHg (second quartile)	In-hospital mortality
Nelskyla *et al.* 2013 [37]	Retrospective analysis of a prospective cohort study, single-center	Australia	Highest PaO_2_	First 24 hours	≥300 mmHg	Not exposed to hyperoxia	In-hospital mortality
***Stroke patients***							
Rincon (a) *et al*. 2014 [19]	Retrospective cohort, multicenter	USA	First PaO_2_	First 24 hours	≥300 mmHg	Normoxia	In-hospital mortality
Young *et al*. 2012 [23]	Retrospective cohort, multicenter	Australia and New Zealand	Worst PaO_2_	First 24 hours	>341 mmHg (upper decile)	Normoxia (PaO_2_ >69 and <341 mmHg, 2nd to 9th deciles)	In-hospital mortality
***Traumatic brain injury***							
Asher *et al*. 2013 [33]	Retrospective cohort, single-center	USA	Highest PaO_2_	First 72 hours	≥200 mmHg	Not exposed to hyperoxia	In-hospital mortality
Brenner *et al*. 2012 [18]	Retrospective cohort, multicenter	USA	Mean PaO_2_	First 24 hours	>200 mmHg	Normoxia	In-hospital mortality
Davis *et al*. 2009 [34]	Retrospective cohort, multicenter	USA	First PaO_2_	On arrival	>487 mmHg	Not exposed to hyperoxia	In-hospital mortality
Raj *et al.* 2013 [22]	Retrospective cohort, multicenter	Finland	Worst PaO_2_	First 24 hours	>100 mmHg	Normoxia	In-hospital mortality
Rincon (b) *et al.* 2014 [38]	Retrospective cohort multicenter	USA	First PaO_2_	First 24 hours	≥300 mmHg	Normoxia	In-hospital mortality

^a^Cutoff used by the reviewers for the analysis. PaO_2_. arterial partial oxygen pressure; ABG, arterial blood gas; ICU, intensive care unit; SpO_2_, peripheral oxygen saturation.

**Table 2:** **Unadjusted and adjusted outcome data extracted for the included studies**

	**Unadjusted data**			**Adjusted data**		
	**Hyperoxia**	**Not exposed to hyperoxia**	**Normoxia**	**OR [95% CI]** ^**a**^	**Comparator group**	**Variables in the model**
***ICU patients***						
de Jonge *et al*. 2008 [16]	NA	NA	NA	1.23 [1.13-1.34] (upper quintile)	PaO_2_ 66-80 mmHg	Age, SAPS II, GCS <15, admission type, individual hospital
Eastwood *et al*. 2012 [20]	58,855/17,225^b^	54,406/22,164	NA	0.73 [0.68-0.78] (upper decile)	PaO_2_ 75-85 mmHg	Site, APACHE III risk of death (O_2_ component removed), FiO_2_, year
Suzuki *et al*. 2013 [2]	21/11	13/6	NA	NA		
Suzuki *et al*. 2014 [32]	35/16	45/9	NA	0.35 [0.12-1.06]	Conservative group	APACHE III score, primary diagnosis, reason for mechanical ventilation
***Post-cardiac arrest***						
Bellomo *et al*. 2011 [21]	531/754	4,609/6,214	1,008/911	1.2 [1.0-1.5]	Normoxia	APACHE III risk of death (O_2_ component removed), treatment limitation, year of admission, lowest glucose in the first 24 h, hospital admission from home, hypoxia/poor O_2_ exchange
Ihle *et al*. 2013 [31]^c^	11/7	137/78	129/60	NA		
Janz *et al*. 2012 [35]^c^	15/31	66/62	NA	1.44 [1.03-2.02]^d^	Not defined	Age, time to return of spontaneous circulation, presence of shock, bystander CPR, initial rhythm
Kilgannon *et al*. 2010 [17]	424/732	2,341/2,829	639/532	1.8 [1.5-2.2]	Not exposed to hyperoxia	Age, ED origin, non-independent functional status at admission, chronic renal failure, active chemotherapy, high heart rate in ICU, hypotension at ICU arrival, hypoxia exposure
Lee *et al*. 2014 [36]^c^	39/14	111/49	NA	0.604 [0.225-1.621]	PaO_2_ 116.9-134.9 mmHg	Age, witness of collapse, hypertension, first monitored rhythm, etiology of cardiac arrest, time to return of spontaneous circulation, time from initiation of therapeutic hypothermia to achieve target temperature, SOFA score (respiratory component removed)
Nelskyla *et al*. 2013 [37]	20/29	24/46	NA	NA		
***Stroke***						
Rincon (a) *et al*. 2014 [19]	182/268	1,252/1,192	582/502	1.22 [1.04-1.48]	Normoxia	Age, GCS, intracranial hemorrhage diagnosis, hypothermia at ICU admission, hypotension or hypertension, organ dysfunctions
Young *et al*. 2012 [23]	101/163	1,028/1,351	927/1,188	NA		
***Traumatic brain injury***						
Asher *et al*. 2013 [33]	87/45	23/38	4/10	3.1 [0.4-24.4] (for survival)	Not exposed to hyperoxia	Age, sex, ISS, politrauma, hematocrit >30%, any PaCO_2_ ≥35 mmHg, chest injury, ARDS in the first 72 h
Brenner *et al*. 2012 [18]	459/207	651/230	587/191	1.56 [1.18-2.07]	Normoxia	Age, sex, ISS, mechanism of injury, admission GCS, admission systolic blood pressure
Davis *et al*. 2009 [34]	210/129	2,342/739	1,602/479	2.0 [1.4-2.7]	Not exposed to hyperoxia	GCS, age, sex, hypotension, ISS, intubation, penetrating mechanism, head Abbreviated ISS, eucapnia, base deficit
Raj *et al*. 2013 [22]	423/144	380/169	270/105	0.94 [0.65-1.36]	Normoxia	APACHE II (O_2_ component removed), year of admission, emergency operation, intracranial pressure monitoring, controlled hypothermia, platelet count
Rincon (b) *et al*. 2014 [38]	176/80	645/311	316/87	1.50 [1.02-2.40]	Not exposed to hyperoxia	Age, comorbidities, GCS, MAP, anemia, organ dysfunction, gender, non-white race, ED boarder status, ICP monitor, abnormal pH, hospital characteristics

Data are expressed as number of survivors/nonsurvivors, unless stated otherwise. ^a^Association between hyperoxia exposure and increased mortality, unless otherwise stated; ^b^PaO_2_ >120 mmHg for hyperoxia; ^c^data requested from the authors; ^d^PaO_2_ as continuous variable. OR, odds ratio; CI, confidence interval; ICU, intensive care unit; *NA,* not available; *SAPS*, Simplified Acute Physiology Score; *GCS*, Glasgow Coma Scale; *APACHE,* Acute Physiology and Chronic Health Evaluation; O_2_, oxygen; FiO_2_, inspired oxygen fraction*; CPR*, cardiopulmonary resuscitation; *ED*, Emergency Department*; SOFA*, Sequential Organ Failure Assessment; ISS, Injury Severity Score; PaCO_2_, partial pressure of carbon dioxide; ARDS, acute respiratory distress syndrome; MAP, mean arterial pressure; *ICP*, intracranial pressure

### Mechanically ventilated ICU patients

The four studies including general populations of mechanically ventilated ICU patients (number of participants (n) = 189,143) were highly heterogeneous in terms of design, outcome measure, criteria for defining hyperoxia exposure, and statistical method applied for analysis (Table 1). de Jonge *et al.* [16] defined the worst PaO_2_ as the one associated with the lowest PaO_2_/FiO_2_. Conversely, Eastwood *et al*. [20] defined the worst PaO_2_ in patients with an FiO_2_ ≥0.5 as that associated with the ABG providing the highest arterial-alveolar (A-a) gradient; for patients with an FiO_2_ <0.5, the lowest PaO_2_ was recorded. If arterial blood gases (ABGs) were taken in patients in whom FiO_2_ <0.5 and ≥0.5 were both recorded during the first 24 hours, the value of PaO_2_ taken when the FiO_2_ ≥0.5 was used. In one study [2] hyperoxia was defined on the basis of a time-weighted SpO_2_ >98%; this parameter was calculated as the mean SpO_2_ between consecutive time points multiplied by the period of time between these time points, with the sum of such time-weighted values being divided by total time to obtain a time-weighted average. For the before-after study by Suzuki *et al.* [32], we considered the ‘conservative’ group as not exposed to arterial hyperoxia and the ‘conventional’ group as exposed to arterial hyperoxia (showing a time-weighted average SpO_2_ of 98.4%).

**Figure 2:**
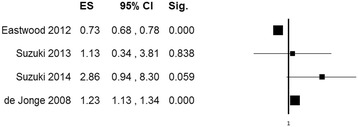
**Forest plot showing individual odds ratios for mortality of studies on general populations of mechanically ventilated ICU patients (k = 4).** Odds ratios >1 (right side of the plot) indicate an association between hyperoxia and higher mortality. Heterogeneity was Q (3) 91.85, *P* <0.001; *I* ^*2*^ = 96.73. The size of the boxes is inversely proportional to the size of the result study variance, so that more precise studies have larger boxes. k, number of studies; ES, effect size; CI, confidence interval; Sig., *P* value.

Extreme heterogeneity was found among the study findings (*Q* (3) = 91.85, *P* <0.001; *I*^*2*^ = 96.73), with an ES ranging from 0.73 to 2.86. Individual ES values (95% CI) are shown in Figure 2. A pooled estimate was not calculated in view of the insufficient homogeneity.

### Patients resuscitated from cardiac arrest

Six retrospective cohort studies (three multicenter [17,21,31], three single-center [35-37]) evaluated patients resuscitated from cardiac arrest (n = 19,144). Different PaO_2_ measures were used to define hyperoxia exposure (Table 1). Most studies used a PaO_2_ cutoff of 300 mmHg [17,21,31,37]. In the study by Janz *et al.* [35], the relationship between mortality and PaO_2_ as a continuous variable was evaluated by multivariable regression analysis; to limit heterogeneity in both hyperoxia definition and analysis between this and the other studies, we reconstructed the unadjusted binary OR by stratifying patients between hyperoxic and nonhyperoxic groups based on a 300 mmHg PaO_2_ cutoff and extracting the number of survivors/nonsurvivors in the two groups. The study by Lee *et al*. [36] was not included in the quantitative synthesis because of the substantial differences found in comparison to the other reported studies (different criteria for defining hyperoxia).

**Figure 3:**
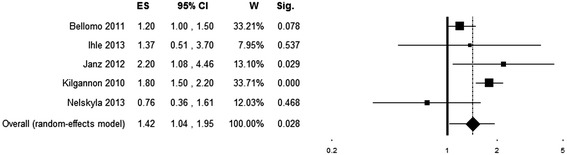
**Forest plot showing individual and pooled odds ratios for mortality of studies on patients resuscitated from cardiac arrest.** Odds ratios >1 (right side of the plot) indicate an association between hyperoxia and higher mortality. Heterogeneity was Q (4) = 12.4, *P* = 0.015; *I* ^*2*^ = 67.73. The size of the boxes is inversely proportional to the size of the result study variance; more precise studies have larger boxes. ES, effect size; CI, confidence interval; W, weight; Sig., *P* value.

The pooled ES shows an association between hyperoxia exposure and increased in-hospital mortality (OR = 1.42 (95% CI 1.04 to 1.92), *P* = 0.028, number of studies (k) = 5), in the presence of a moderately high heterogeneity (*Q* (4) = 12.4, *P* = 0.015; *I*^*2*^ = 67.73) (Figure 3). A funnel plot indicates no obvious publication bias (Additional file 3).

Results of the moderator analyses are shown in Table 3. A significant overall ES was found among multicenter (k = 3) but not single-center (k = 2) studies. A significant association with mortality was indicated only in the study that used the first available PaO_2_. An association of borderline statistical significance was shown only for studies in which adjusted data were used. Meta-regression analyses showed a progressively weaker association with increasing prevalence of chronic cardiovascular disease (k = 4, range 12 to 36%). No significant moderator effect was shown by the following variables: mean age (k = 5; range 60.5 to 64 years); gender (k = 4; range 54 to 80% of males); hospital mortality (k = 5; range 32.4 to 66%); average delay to return of spontaneous circulation (k = 3; range 15 to 25.7 minutes); prevalence of initial shockable rhythm (ventricular tachycardia/fibrillation, k = 3; range 40 to 100%); prevalence of out-of-hospital cardiac arrest (k = 5; range 43 to 100%). Study quality as defined by the NOS score had no effect on the ES.

**Table 3:** **Moderator analyses for studies on patients resuscitated from cardiac arrest (k = 5)**

	***k***	**Effect size (OR)**	**95% CI**	***P***	***Q***	***I*** ^***2***^	***P*** ^**a**^
***Study design***							0.836
Multicenter	3	1.46	1.03-2.07	0.031	8.15^*^	75.45	
Single-center	2	1.30	0.46-3.70	0.622	4.10^*^	75.60	
***Hyperoxia, definition***							0.017
First PaO_2_	1	1.80	1.50-2.20	0.000	-	-	
Highest PaO_2_	2	1.30	0.46-3.70	0.622	4.10^*^	75.60	
Worst PaO_2_	2	1.21	0.99-1.47	0.064	0.06	0.00	
***Comparator group***							0.381
Not exposed to hyperoxia	3	1.54	0.93-2.54	0.095	5.23	61.78	
Normoxia	2	1.21	0.99-1.47	0.064	0.06	0.00	
***Outcome data***							0.791
Adjusted	2	1.47	0.99-2.19	0.057	8.12^**^	87.69	
Unadjusted	3	1.32	0.68-2.58	0.409	4.10	51.21	
	***k***	***Beta***	***P***				
***Study quality***							
NOS score	5	−0.24	0.184				
***Chronic cardiovascular disease***							
Percentage	4	−0.04	0.024				

^a^Analysis of variance Q-test between study subgroups. ^*^ *P* <0.05; ^**^ *P* <0.01. k, number of studies; OR, odds ratio; CI, confidence interval; Q, test for heterogeneity; I^2^, inconsistency between studies; PaO_2_, arterial partial oxygen pressure; Beta, coefficient of the random effects meta-regression analysis: positive and negative values indicate direct and inverse relationships, respectively; NOS Newcastle-Ottawa Scale.

### Patients with stroke

**Figure 4:**
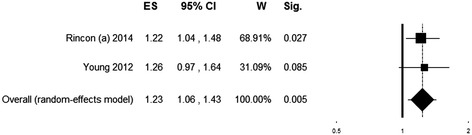
**Forest plot showing individual and pooled odds ratios for mortality of studies on patients with stroke.** Odds ratios >1 (right side of the plot) indicate an association between hyperoxia and higher mortality. Heterogeneity was Q (1) = 0.04, *P* = 0.844, *I* ^*2*^ = 0. The size of the boxes is inversely proportional to the size of the result study variance, so that more precise studies have larger boxes. ES, effect size; CI, confidence interval; W, weight; Sig., *P* value.

Two multicenter retrospective cohort studies [19,23] evaluated the relationship between in-hospital mortality and exposure to arterial hyperoxia in the first 24 hours of ICU admission in patients with stroke (*n* = 5,537). Rincon *et al.* [19] defined patients with the first PaO_2_ ≥300 mmHg as being exposed to hyperoxia. Young *et al.* [23] evaluated the independent association between mortality and deciles of PaO_2_, with the upper decile (PaO_2_ >341 mmHg) used as the reference category. To make the two studies comparable, for [23] we considered patients in the upper decile as those being exposed to hyperoxia and reconstructed the unadjusted OR for mortality; patients in the first decile (PaO_2_ ≤69 mmHg) were excluded. The pooled ES indicates an association between hyperoxia exposure and increased hospital mortality (OR = 1.23 (95% CI 1.06 to 1.43), *P* = 0.005; *Q* (1) = 0.04, *P* = 0.844, *I*^*2*^ = 0) (Figure 4).

### Patients with traumatic brain injury

Four multicenter [18,22,34,38] and one single-center [33] retrospective cohort studies evaluated patients with traumatic brain injury (n = 7,488). Different criteria were used to define hyperoxia in terms of time of assessment, PaO_2_ selection and cutoff value used (Table 1). All studies reported the adjusted ORs for hospital mortality (Table 2).

**Figure 5:**
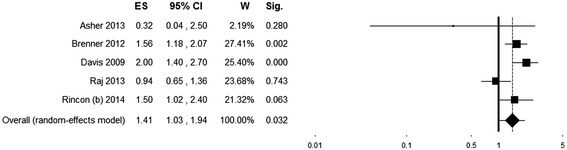
**Forest plot showing individual and pooled odds ratio for mortality of studies on patients with traumatic brain injury.** Odds ratios >1 (right side of the plot) indicate an association between hyperoxia and higher mortality. Heterogeneity was Q (4) = 11.28, *P* = 0.024; *I* ^*2*^ = 64.54. The size of the boxes is inversely proportional to the size of the result study variance; more precise studies have larger boxes. ES, effect size; CI, confidence interval; W, weight; Sig., *P* value.

The pooled ES indicates an association between hyperoxia exposure and increased mortality (OR = 1.41 (95% CI 1.03 to 1.94), *P* = 0.032) in the presence of significant heterogeneity (*Q* (4) = 11.28, *P* = 0.024; *I*^*2*^ = 64.54) (Figure 5). The funnel plot indicated no obvious publication bias (Additional file 4). The exclusion of the study by Asher *et al.* [33] (single-center, time of PaO_2_ assessment beyond the first 24 hours) in the sensitivity analysis did not substantially change the combined ES (OR = 1.46 (95% CI 1.08 to 1.98)) nor did it decrease heterogeneity (*I*^*2*^ = 67.29). Results of the moderator analyses are shown in Table 4, with studies stratified based on design, PaO_2_ value and cutoff used for defining hyperoxia, time of PaO_2_ assessment and comparator group. Study quality as defined by the NOS score, mean age and gender did not influence the ES.

**Table 4:** **Moderator analyses for studies in patients with traumatic brain injury (k = 5)**

	***k***	**Effect size (OR)**	**95% CI**	***P***	***Q***	***I*** ^***2***^	***P*** ^**a**^
***Study design***							0.154
Multicenter	4	1.46	1.08-1.98	0.014	9.17^*^	67.29	
Single-center	1	0.32	0.04-2.50	0.280	-	-	
***Hyperoxia, definition***							0.019
First PaO_2_	2	1.79	1.36-2.36	0.000	1.09	8.51	
Highest PaO_2_	1	0.32	0.04-2.50	0.280	-	-	
Mean PaO_2_	1	1.56	1.18-2.07	0.002	-	-	
Worst PaO_2_	1	0.94	0.65-1.36	0.743	-	-	
***Hyperoxia, time of assessment***							0.154
First 24 hours	4	1.46	1.08-1.98	0.014	9.17^*^	67.29	
Beyond the first 24 hours	1	0.32	0.04-2.50	0.280	-	-	
***PaO***_***2***_ ***cutoff***							0.126
<300 mmHg (moderate hyperoxia)	3	1.14	0.68-1.90	0.618	6.32^*^	68.34	
≥300 mmHg (extreme hyperoxia)	2	1.79	1.36-2.36	0.000	1.09	8.51	
***Comparator group***							0.824
Not exposed to hyperoxia	2	1.08	0.20-5.89	0.933	2.94	66.03	
Normoxia	3	1.31	0.95-1.81	0.101	4.93	59.47	
	***k***	***Beta***	***P***				
***Study quality***							
NOS score	5	0.26	0.281				

^a^Analysis of variance Q test between study subgroups. ^*^ *P* <0.05. k, number of studies; OR, odds ratio; CI, confidence interval; Q, test for heterogeneity; I^2^, inconsistency between studies; PaO_2_, arterial partial oxygen pressure; Beta, coefficient of the random effects meta-regression analysis: positive and negative values indicate direct and inverse relationships, respectively; NOS, Newcastle-Ottawa Scale.

## Discussion

The main findings of our systematic review and meta-analysis may be summarized in three points. First, the majority of studies that explored the relationship between arterial hyperoxia and mortality were retrospective observational investigations, with only one interventional prospective before-after study comparing a conventional ventilation strategy against a more conservative strategy. RCTs comparing two different PaO_2_/SpO_2_-targeted ventilation strategies are lacking. Second, high heterogeneity was found between studies in the criteria used for defining hyperoxia exposure (PaO_2_ value and cutoff selected, time of assessment) and the statistical method applied for analysis; this limits comparison between the study results. Third, while studies in general populations of ICU patients gave highly inconsistent results, an association between arterial hyperoxia and increased hospital mortality was found in critically ill patient subsets (post-cardiac arrest, stroke, traumatic brain injury).

Four studies evaluated general populations of mechanically ventilated patients [2,16,20,32] and gave highly inconsistent results. Two large multicenter retrospective studies [16,20] reported a U-shaped relationship between PaO_2_ levels and mortality by unadjusted analysis. After adjusting for potential confounders including severity of illness, the association between higher PaO_2_ levels and mortality was confirmed only by de Jonge *et al.* [16], while Eastwood *et al.* [20] showed a protective effect of hyperoxia. Differences in the methods applied for the analysis (PaO_2_ stratified in quintiles/deciles, different reference categories) make these studies difficult to compare. A pilot before-after trial was the only interventional study that compared conventional management to a conservative strategy using an SpO_2_ target between 90 and 92% [32]. Although this study was underpowered to demonstrate a difference in mortality, it supported the feasibility and safety of a restrictive O_2_ therapy, which led to a marked reduction in O_2_ exposure without being associated with major clinical and physiological adverse effects.

Our analysis showed a significant association between hyperoxia exposure and mortality in patients resuscitated from cardiac arrest. This is consistent with the result of a recent meta-analysis by Wang *et al.* [39]. In a meta-analysis of animal trials, the administration of 100% O_2_ therapy following resuscitation from cardiac arrest was associated with worse neurological outcomes as compared with lower O_2_ concentrations [40]. An association between hyperoxia exposure and poor neurological outcomes has been reported by several authors [17,35,36,41], but not confirmed by a recent multicenter observational study [42] that instead highlighted PaCO_2_ as a possible confounding factor. The adverse effects of hyperoxia may be due to enhanced oxidative stress, which may be particularly deleterious during the early reperfusion phase after cardiac arrest, and a vasoconstrictor effect that may paradoxically lead to a net reduction in local O_2_ delivery to tissues including the myocardium and brain [43]. Mechanisms by which hyperoxia causes vasoconstriction include an inhibition of vasodilator (prostaglandins, nitric oxide) by reactive O_2_ species [43]. Of note, the strength of association between hyperoxia and mortality was inversely related to the prevalence of chronic cardiovascular disease. We speculate that the response to a high O_2_ tension may be blunted in the presence of an underlying endothelial dysfunction, where nitric oxide levels may be chronically low [44]. However, this hypothesis can be challenged by data showing a deleterious effect of high O_2_ in patients with severe coronary artery disease and myocardial infarction [45,46]. An alternative hypothesis may be that hyperoxia exposure had a minor role on mortality when the prevalence of cardiovascular comorbidity was higher.

The exposure to arterial hyperoxia in the first 24 hours of ICU admission was associated with higher mortality in patients with stroke, although this result is limited by the low number of studies included. Previous RCTs showed only transient radiological (magnetic resonance imaging) and clinical improvement [47], no benefit [48] or worse outcomes [49] in stroke patients receiving supplemental O_2_ during their initial management. The Stroke Oxygen Study RCT, due to report in early 2016, is comparing three-day continuous or nocturnal O_2_ administration with no supplemental O_2_ in 6,000 patients (ISRCTN52416964, www.controlled-trials.com). Likewise, arterial hyperoxia was associated with increased hospital mortality in patients with traumatic brain injury. This should, however, be interpreted with caution given the heterogeneous characteristics of the studies included. The rationale for giving high O_2_ concentrations to patients with traumatic brain injury is to improve brain O_2_ delivery and metabolism [50]. While studies using indirect measures of brain metabolism provided promising results [7], these were not subsequently confirmed by a study that found no improvement in brain O_2_ utilization measured by positron emission tomography one hour after ventilation with 100% O_2_ [51].

The different criteria used for defining hyperoxia exposure were the main source of heterogeneity among the analyzed studies. The selection of ‘worst’ PaO_2_ based on the A-a gradient [20-23,31] has been questioned by several authors, as this gradient does not correlate in a linear fashion with PaO_2_ as the FiO_2_ increases [35] and this method may reduce the probability of finding any association between hyperoxia and mortality [52]. However, a subanalysis by Bellomo *et al.* [21] on a sample of 100 patients showed that the worst PaO_2_ was more representative of mean PaO_2_ than was the first PaO_2_ measured after ICU admission. Different PaO_2_ measures may have different pathophysiological consequences. If even short periods of hyperoxia were dangerous, then the highest PaO_2_ would represent the most sensitive approach to identify patients at risk; conversely, if the overall effect depended on the total amount of excess O_2_ received, then the mean PaO_2_ or a time-weighted measure would be a better choice. Alternatively, the first PaO_2_ measurement would be preferable if the deleterious effects of hyperoxia were more pronounced in the early phase of the disease. Regardless of disease category, all studies that considered the first available PaO_2_ [17,19,38] found an independent association between hyperoxia exposure and hospital mortality, while studies using other measures showed more inconsistent results. This may suggest that exposure to high O_2_ tensions in the early phase of the critical illness may be particularly associated with worse outcomes. Interestingly, in a subgroup analysis by de Jonge *et al.* [16], exposure to hyperoxia as defined by higher mean PaO_2_ values during the entire ICU stay was not independently associated with mortality. All studies that considered a timespan longer than the first 24 hours for assessing oxygenation status did not find any significant association between arterial hyperoxia and worse outcomes [2,33,36].

In most of the studies, patients were categorized as hyperoxic or nonhyperoxic based on an arbitrarily predetermined PaO_2_ cutoff value. Similarly, our analysis was based on binary ORs of hyperoxia exposure versus nonexposure. This approach may be limited by poor resolution in describing the relationship between increasing arterial O_2_ tensions and outcome. Several studies in which PaO_2_ was analyzed as a continuous variable showed a linear relationship between increasing arterial O_2_ tensions and mortality, without a clear threshold for harm [30,35]. Furthermore, there is no consensus on the PaO_2_ cutoff value to use for defining hyperoxia exposure, which varied markedly across the analyzed studies. This is likely to influence the associations observed. In a meta-regression analysis on the overall set of studies, the association between hyperoxia and worse outcome appeared to become stronger when the PaO_2_ cutoff value used for defining exposure increased (data shown in Additional file 5).

### Strengths and weaknesses

This is the first systematic review on the relationship between arterial hyperoxia and mortality in critically ill patients that gathers together data from a large number of subjects within several distinct disease categories. In our quantitative data syntheses, every effort was made to control for possible sources of heterogeneity and confounding factors. The authors were contacted if additional unpublished data were needed; any overlap between study populations was avoided. Whenever possible, adjusted outcome data were used and/or hypoxic patients excluded. Moderator analyses were performed to analyze the impact of several sources of heterogeneity (definition of hyperoxia exposure, study design). Study quality was assessed by means of a standardized scale and its impact on the studied association was explored. A random-effects model was used to pool data to account for unmeasured confounding factors and sources of heterogeneity.

Our analysis has several limitations. First, the studies were mainly observational investigations that cannot directly support any causal relationship between hyperoxia exposure and worse outcome. Higher PaO_2_ levels may simply reflect the clinicians’ attempts to optimize O_2_ delivery by administering a higher FiO_2_; thus PaO_2_ becomes a marker of illness severity rather than being directly responsible for the outcome. Second, the included studies used different criteria for defining hyperoxia exposure and applied different statistical methods for analysis (PaO_2_ as an ordinal/continuous variable; different multivariable regression models). Third, hypoxemic patients could not always be excluded from the analysis: these patients are likely to be responsible for an increased mortality in the subgroup of those not exposed to hyperoxia and might thus have blunted the studied association. Finally, we did not include unpublished studies, dissertations, or conference abstracts. We decided to consider only published material to ensure that only higher quality, peer-reviewed studies were included in the analysis.

### Clinical implications and directions for future research

Given the widespread use of O_2_ therapy in critical care, clinicians should be aware of the potentially deleterious effects of excessive O_2_ administration. Several studies reported that FiO_2_ is rarely adjusted for arterial hyperoxia, especially when this occurs at lower FiO_2_ settings [1,2]. The rationale for giving supplemental O_2_ to nonhypoxemic patients should be reconsidered as there is insufficient evidence of benefit [43]. When hemoglobin is fully saturated, additional O_2_ only marginally increases O_2_ transport capacity; conversely, a paradoxical decrease in regional O_2_ delivery could be caused by vasoconstriction [53].

An urgent need exists for adequately designed studies to provide conclusive answers regarding the safety of hyperoxia in critically ill patients. Only RCTs can confirm a causal relationship between hyperoxia exposure and higher mortality. These trials should evaluate ventilation strategies using different PaO_2_ targets for titrating FiO_2_, rather than comparing two arbitrarily selected FiO_2_ targets. A pilot before-after study has supported the safety and feasibility of a conservative oxygen therapy [32]. RCTs comparing current liberal ventilation practices to more restrictive approaches in ICU patients are currently ongoing (ClinicalTrials.gov, NCT01319643 and NCT01722422).

There is an imperative to identify the best criteria that define hyperoxia exposure in observational studies. The relationship between hyperoxia and mortality should be evaluated using different criteria for defining hyperoxia exposure, comparing different PaO_2_ measures and the time of assessment. This analysis may have important pathophysiological implications and could clarify whether early exposure to hyperoxia during critical illness is more deleterious. In future research, it would also be more useful to assess and report the relationship between mortality and PaO_2_ as a continuous variable, instead of stratifying patients on the basis of an arbitrary PaO_2_ cutoff value. Finally, further studies should address other specific categories of critically ill patients, such as sepsis, polytrauma, post-operative cases and hemorrhagic shock.

## Conclusions

The majority of studies that have explored the relationship between arterial hyperoxia and mortality in critically ill patients are retrospective observational investigations, with only one prospective before-after study supporting the safety of a more conservative strategy. A quantitative data synthesis was not possible for studies on general populations of mechanically ventilated ICU patients because of differences in design, definition of hyperoxia, and the outcome measure reported. Hyperoxia exposure may be associated with mortality in patient subsets (post-cardiac arrest, stroke and traumatic brain injury). However, these results must be interpreted cautiously given the heterogeneity in criteria used for defining hyperoxia exposure and a significant inconsistency between study findings. Nevertheless, these data provide the rationale for future RCTs comparing conventional practice against more restrictive oxygenation targets.

## Key messages

- There is insufficient evidence regarding the safety of arterial hyperoxia in critically ill patients. Most of the existing studies are observational investigations with highly heterogeneous characteristics and inconsistent results. Randomized controlled trials are lacking.
- Arterial hyperoxia may be associated with higher mortality in some critically ill patient subsets (post-cardiac arrest, stroke and traumatic brain injury).

## Additional files

Additional file 1:
**Search strategy.** Search strategy applied for Medline (PubMed) and adapted for the other electronic databases. Additional file 2:
**Study quality assessment with the Newcastle-Ottawa Scale (NOS).** Items fulfilled are indicated by an ‘*’. Additional file 3:
**Funnel plot related to the five studies evaluating patients resuscitated from cardiac arrest.** Funnel plot analysis did not show any asymmetry. Additional file 4:
**Funnel plot related to the five studies evaluating patients with traumatic brain injury.** Funnel plot analysis did not show any asymmetry. Additional file 5:
**Meta-regression analysis showing the impact on the study ES of the PaO**_**2**_ **cutoff used for defining hyperoxia.** Each circle represents a study. The size of the circles is inversely proportional to the size of the result study variance, so that more precise studies have larger circles. Meta-regression analysis showed that the strength of the association between arterial hyperoxia and mortality increased with increasing PaO_2_ cutoff values used for defining hyperoxia exposure.

## Abbreviations

(A-a): alveolar-arterial
ABG: arterial blood gas
ALI: acute lung injury
APACHE: Acute Physiology and Chronic Health Evaluation
CI: confidence interval
COPD: chronic obstructive pulmonary disease
ES: effect size
FiO_2_: inspired oxygen fraction
I^2^: inconsistency analysis
ICU: intensive care unit
ISS: Injury Severity Score
k: number of studies
n: number of participants
NOS: Newcastle-Ottawa Scale
O_2_: oxygen
OR: odds ratio
PaO_2_: arterial partial oxygen pressure
RCT: randomized controlled trial
SAPS: Simplified Acute Physiology Score
SOFA: Sequential Organ Failure Assessment
SpO_2_: peripheral oxygen saturation
