# Supplementary material for: Arterial hyperoxia and mortality in critically ill patients: a systematic review and meta-analysis
Source: Crit Care. 2014 Dec 23;18(6):711. doi: 10.1186/s13054-014-0711-x (PMC4298955; doi:10.1186/s13054-014-0711-x)
Supplement: Additional file 1: — Search strategy. Search strategy applied for Medline (PubMed) and adapted for the other electronic databases. [file 13054_2014_711_MOESM1_ESM.pdf]

Additional file 1 – Search strategy

PubMed

01- Hyperoxia.ti,ab.
02- Hyperoxia.tw.
03- Hyperoxemia.ti,ab.
04- Hyperoxemia.tw.
05- Arterial oxygen.ti,ab.
06- High oxygen.ti,ab.
07- Arterial blood gas.ti,ab.
08- Oxygen saturation.ti,ab.
09- 1 or 2 or 3 or 4 or 5 or 6 or 7 or 8
10- Critically ill.ti,ab.
11- Acutely ill.ti,ab.
12- Intensive care.ti,ab.
13- Critical care.ti,ab.
14- 10 or 11 or 12 or 13
15- Mechanically ventilated.ti,ab.
16- Cardiac arrest.ti,ab.
17- Cardiopulmonary resuscitation.ti,ab.
18- CPR.ti,ab.
19- Heart arrest.ti,ab.
20- 16 or 17 or 18 or 19
21- Traumatic brain injury.ti,ab.
22- Head trauma.ti,ab.
23- 21 or 22
24- Stroke.ti,ab.
25- Intracranial bleeding.ti,ab.
26- Intracranial hemorrhage.ti,ab.
27- 24 or 25 or 26
28- Sepsis.ti,ab.
29- Septic shock.ti,ab.
30- 28 or 29
31- Trauma.ti,ab.
32- Post operative.ti,ab.
33- Post-operative.ti,ab.
34- Post surgery.ti,ab.
35- Post-surgery.ti,ab.
36- Elective surgery.ti,ab.
37- 32 or 33 or 34 or 35 or 36
38- Cardiac failure.ti,ab.
39- Heart failure.ti,ab.
40- Myocardial infarction.ti,ab.
41- Shock.ti,ab.
42- 14 or 20 or 23 or 27 or 30 or 31 or 37 or 38 or 39 or 40 or 41
43- Mortality.tw.

44- Outcome.tw.
45- Death.tw.
46- Survival.tw.
47- 43 or 44 or 45 or 46
48- 9 and 14 and 47
49- 9 and 42 and 47
