# Supplementary material for: Arterial hyperoxia and mortality in critically ill patients: a systematic review and meta-analysis
Source: Crit Care. 2014 Dec 23;18(6):711. doi: 10.1186/s13054-014-0711-x (PMC4298955; doi:10.1186/s13054-014-0711-x)
Supplement: Additional file 2: — Study quality assessment with the Newcastle-Ottawa Scale (NOS). Items fulfilled are indicated by an ‘*’. [file 13054_2014_711_MOESM2_ESM.pdf]

Additional File 2 – Study quality assessment with the Newcastle-Ottawa Scale (NOS).

	Representativeness of the exposed cohort	Selection of the non-exposed cohort	Ascertainment of exposure	Demonstration that outcome was not present at start	Comparability of cohorts - Illness severity	Comparability of cohorts - FiO_2	Assessment of outcome	Follow-up long enough	Completeness of follow-up (lost to follow-up <10%)	Total NOS score
ICU patients										
de Jonge 2008 [16]	exclusions for missing data not stated	*	*	*	* (adjusted)		*	*	*	7
Eastwood 2012 [20]	exclusions for missing data 22%	*	*	*	* (adjusted)	* (adjusted)	*	*	*	8
Suzuki 2013 [2]	exclusions for missing data not stated	*	*	*	* (balanced)		*	*	*	7
Suzuki 2014 [32]	*	*	*	*	* (adjusted)	* (balanced at admission)	*	*	*	9
Post-cardiac arrest										
Bellomo 2011 [21]	*	*	*	*	* (adjusted)		*	*	*	8
Ihle 2013 [31]	exclusions for missing data 39%	*	*	*	adjusted data not available for cohort 2010-2011	adjusted data not available for cohort 2010-2012	*	*	*	6
Janz 2012 [35]	*	*	*	*			*	*	*	7
Kilgannon 2010 [17]	exclusions for missing data 27.6%	*	*	*			*	*	*	6
Lee 2014 [36]	*	*	*	*	* (adjusted)		*	*	*	8
Nelskyla 2013 [37]	*	*	*	*	* (balanced)		*	*	*	8
Stroke										
Rincon (a) 2014 [19]	exclusions for missing data not stated	*	*	*	* (adjusted)		*	*	*	7
Young 2012 [23]	exclusions for missing data 16%	*	*	*	* (adjusted)	* (adjusted)	*	*	*	8
Traumatic brain injury										

Asher 2013 [33]	exclusions for missing data not stated	*	*	*	* (adjusted)		*	*	*	7
Brenner 2012 [18]	exclusions for missing data not stated	*	*	*	* (adjusted)		*	*	*	7
Davis 2009 [34]	*	*	*	*	* (adjusted)		*	*	*	8
Raj 2013 [22]	exclusions for missing data 14%	*	*	*	* (adjusted)		*	*	exclusions for missing mortality data 12.5%	6
Rincon (b) 2014 [38]	exclusions for missing data 30.8%	*	*	*			*	*	*	6

*Item fulfilled
