# Supplementary figures and images for: Arterial hyperoxia and mortality in critically ill patients: a systematic review and meta-analysis
Source: Crit Care. 2014 Dec 23;18(6):711. doi: 10.1186/s13054-014-0711-x (PMC4298955; doi:10.1186/s13054-014-0711-x)

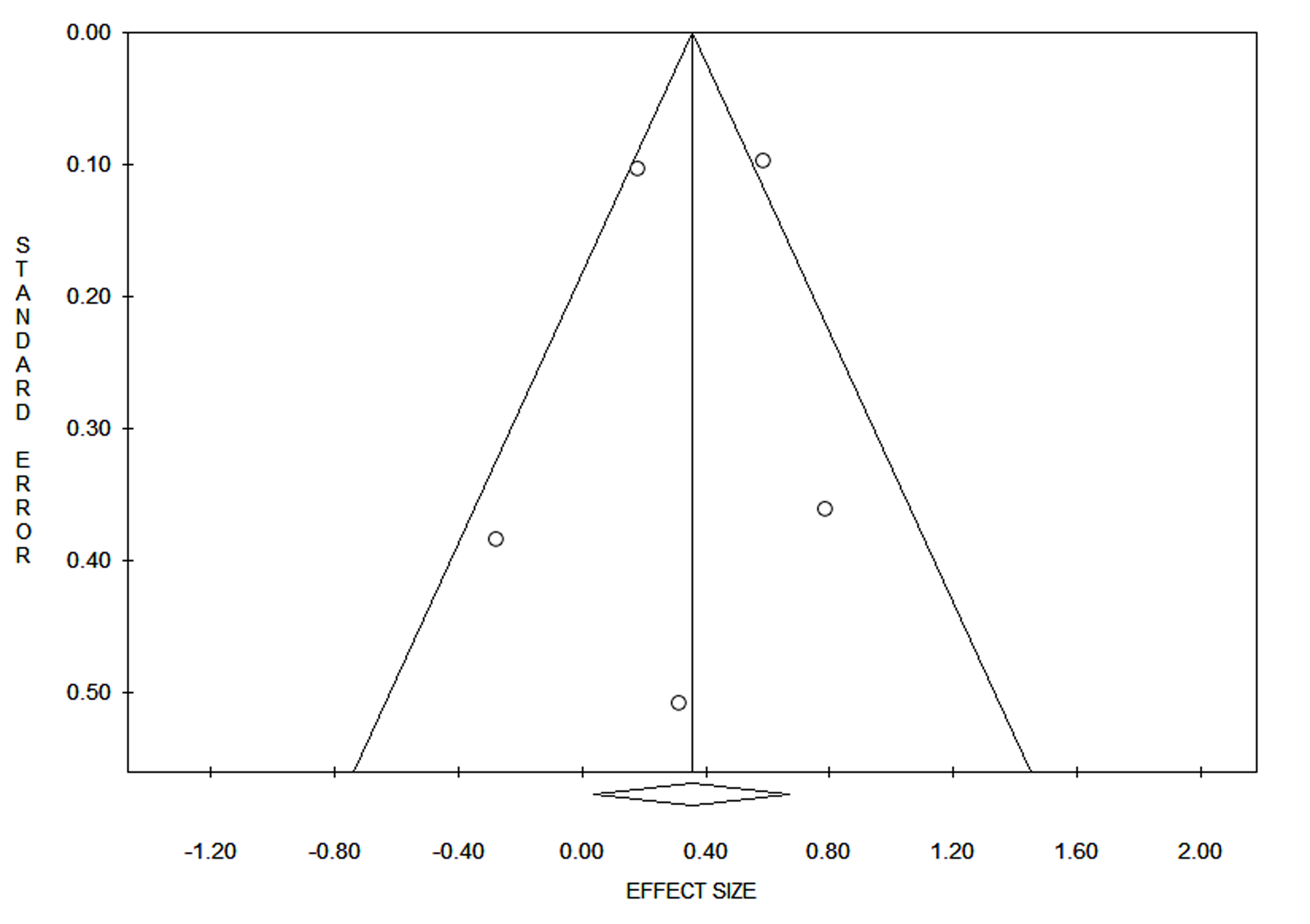

Supplement: Additional file 3: — Funnel plot related to the five studies evaluating patients resuscitated from cardiac arrest. Funnel plot analysis did not show any asymmetry. [file 13054_2014_711_MOESM3_ESM.tiff]

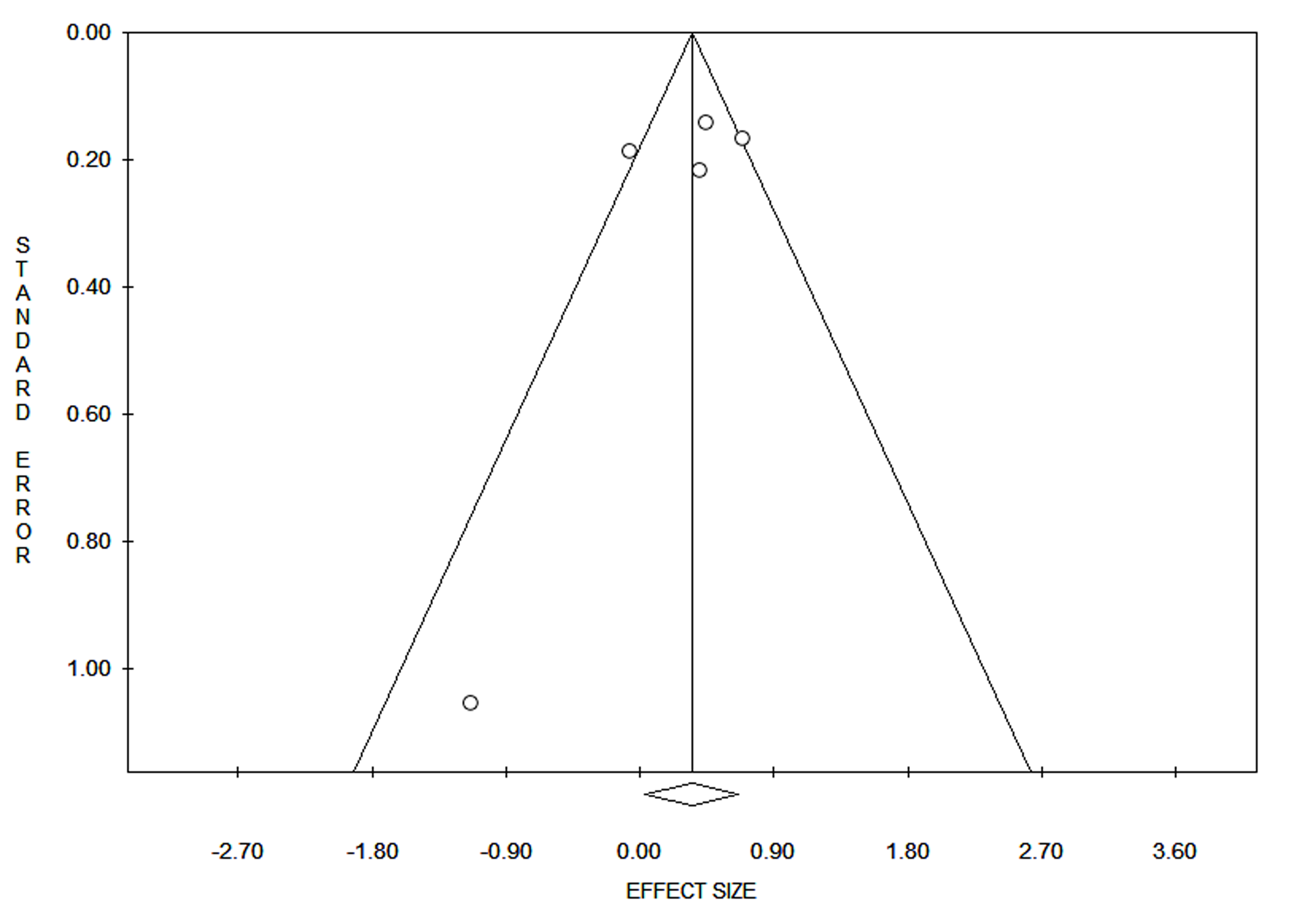

Supplement: Additional file 4: — Funnel plot related to the five studies evaluating patients with traumatic brain injury. Funnel plot analysis did not show any asymmetry. [file 13054_2014_711_MOESM4_ESM.tiff]

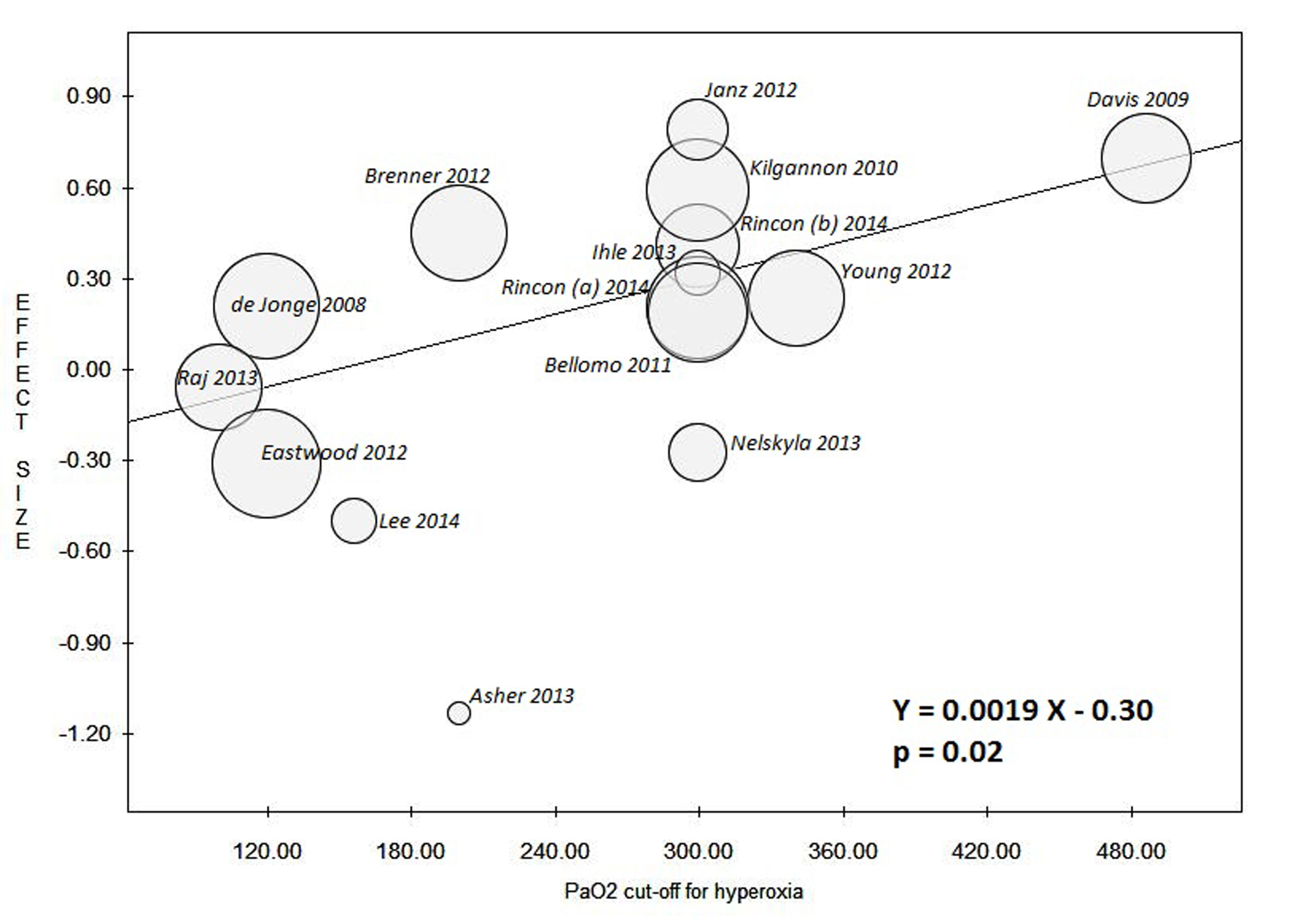

Supplement: Additional file 5: — Meta-regression analysis showing the impact on the study ES of the PaO 2 cutoff used for defining hyperoxia. Each circle represents a study. The size of the circles is inversely proportional to the size of the result study variance, so that more precise studies have larger circles. Meta-regression analysis showed that the strength of the association between arterial hyperoxia and mortality increased with increasing PaO2 cutoff values used for defining hyperoxia exposure. [file 13054_2014_711_MOESM5_ESM.tiff]
